# Interstitial Glucose Metabolism Monitoring as an Additional Method for Objective Assessment of Donor Liver, Prediction and Immediate Diagnosis of Early Graft Dysfunction

**DOI:** 10.17691/stm2022.14.3.04

**Published:** 2022-05-28

**Authors:** A.I. Sushkov, S.E. Voskanyan, V.S. Rudakov, M.V. Popov, K.K. Gubarev, D.S. Svetlakova, A.I. Artemiev

**Affiliations:** Head of the Laboratory of Advanced Surgical Technologies; Burnasyan Federal Medical Biophysical Center of FMBA of Russia, 23 Marshal Novikov St., Moscow, 123098, Russia; Professor, Corresponding Member of the Russian Academy of Sciences, Deputy Chief Physician for Surgical Care — Head of the Center for Surgery and Transplantology; Burnasyan Federal Medical Biophysical Center of FMBA of Russia, 23 Marshal Novikov St., Moscow, 123098, Russia; Surgeon, Surgery Department for Human Organ and (or) Tissue Donation Coordination; Burnasyan Federal Medical Biophysical Center of FMBA of Russia, 23 Marshal Novikov St., Moscow, 123098, Russia; Junior Researcher, Laboratory of Advanced Surgical Technologies; Burnasyan Federal Medical Biophysical Center of FMBA of Russia, 23 Marshal Novikov St., Moscow, 123098, Russia; Head of the Surgery Department for Human Organ and (or) Tissue Donation Coordination; Burnasyan Federal Medical Biophysical Center of FMBA of Russia, 23 Marshal Novikov St., Moscow, 123098, Russia; Surgeon, Surgery Department for Human Organ and (or) Tissue Donation Coordination; Junior Researcher, Laboratory of Advanced Surgical Technologies; Burnasyan Federal Medical Biophysical Center of FMBA of Russia, 23 Marshal Novikov St., Moscow, 123098, Russia; Head of Surgery Department No.2; Burnasyan Federal Medical Biophysical Center of FMBA of Russia, 23 Marshal Novikov St., Moscow, 123098, Russia

**Keywords:** liver transplantation, early allograft dysfunction, primary non-function graft, static cold storage, microdialysis

## Abstract

The current clinical practice of assessing the quality and suitability of a donor liver for human transplantation does not exclude cases of primary graft dysfunction of the transplanted organ and, at the same time, leads to an unreasonable refusal to transplant a significant number of functionally suitable organs. In this regard, searching for new methods for additional objective assessment and monitoring of the state of donor organs in the peritransplant period is relevant.

**The aim of the study** was to determine the clinical utility of monitoring interstitial concentrations of glucose and its metabolites to assess the viability and functional state of a donor liver before and after human transplantation.

**Materials and Methods:**

A retrospective observational single-center study included 32 cases of liver transplantation. Along with standard methods for assessing the initial function of grafts during the first week after surgery, interstitial (in the transplanted liver) concentrations of glucose and its metabolites were monitored. In 18 cases, the interstitial glucose metabolism was also studied during static cold storage (SCS).

**Results:**

With the development of early allograft dysfunction (EAD), compared with the uneventful post-transplant period, statistically significantly higher interstitial lactate concentrations were observed as early as 3 h after reperfusion: 12.3 [10.1; 15.6] mmol/L versus 7.2 [3.9; 9.9] mmol/L (p=0.003). A value above 8.8 mmol/L may be considered as a criterion for the immediate diagnosis of EAD (sensitivity — 89%, specificity — 65%).

Interstitial lactate concentration at the end of SCS and the area under the “lactate concentration–SCS duration” curve were associated with the initial graft function. Values of these parameters greater than 15.4 mmol/L and 76.1 mmol/L·h, respectively, with a sensitivity of 100% in both cases and a specificity of 77 and 85%, may be used to assess the risk of primary EAD.

**Conclusion:**

Monitoring of interstitial concentrations of glucose and its metabolites, primarily, lactate, is an objective additional method for the assessment of the donor liver viability both during SCS and in the early postoperative period.

## Introduction

Over the past two decades, the number of liver transplants performed annually in Russia has increased almost fiftyfold: from 12 operations in 2000 to 584 in 2019 [1]. Despite the impressive growth rates of this type of medical care, it is not enough to meet the demand of the country’s population. Thus, according to the International Registry in Organ Donation and Transplantation, in 2020, the number of deceased-donor liver transplants per one million in the USA, Spain, Portugal, Italy, Croatia, and France exceeded 20 procedures, while in the Russian Federation this figure was 3.04 [2]. Further growth in the number of transplants certainly depends on the creation of new and development of existing donation programs in all regions of the country. Moreover, an important area will be an increased efficiency of using the existing donor resource, i.e. maximizing the practice of multi-organ procurement. Refusal to retrieve the liver from a deceased donor for subsequent transplantation may be due to medical grounds (prolonged episodes of asystole or unstable hemodynamics, requiring the infusion of high doses of inotropic and vasopressor drugs; inappropriate results of laboratory and instrumental tests, visual and histological evaluation of the organ); technical aspects of procurement itself (poor quality of perfusion with a preservation solution, damage to important anatomical structures); the lack of demand for organs in the clinics closest to the donor hospital and the impossibility of their transport to the transplantation center where it could be transplanted without exceeding the deadlines for preservation. The lack of generalized and regularly updated information on the reasons for refusals from the removal and/or transplantation of the liver and other organs from effective deceased donors does not allow to spot their real frequency and structure of reasons for refusal. According to the OPTN/ SRTR [3] and Eurotransplant [4] registries for 2019, the proportion of effective deceased donors, from whom a liver was procured for transplantation, in the United States was 77% (9151 organs from 11,870 donors), and in the Eurotransplant zone — 75% (1536 organs from 2042 donors). According to the register of the Russian Transplant Society, in 2019, the frequency of deceased-donor liver utilization was 60% (437 organs from 732 donors), and during 2015–2018 it was in the range from 44 to 54% [1]. Thus, even with the current level of donor activity, the number of deceased-donor liver transplants in Russia may be increased by 70– 100 per year.

Obviously, the extension of the criteria for a deceased donor liver suitability for transplantation, on the one hand, increases the number of procedures performed and reduces mortality among transplant candidates, and, on the other hand, it increases the risk of primary non-function or early allograft dysfunction (EAD) after transplantation, which affects the immediate and long-term outcomes of the operations and increases the need for repeated transplantations.

Current clinical practice involves the assessment of the quality of a donor organ and the feasibility of its subsequent transplantation based on the results of a number of standard laboratory and instrumental tests of a potential donor (a person with an established diagnosis of brain death) before the procurement, as well as during the surgery based on the subjective visual assessment. In some cases, an urgent histological study is acceptable, which, however, is not common due to the possible increase in the time of graft preservation and only an indirect assessment of the functional suitability of the organ. The existing approaches allow for the risks of primary non-function (PNF) graft and at the same time lead to unreasonable refusal to transplant a significant number of organs, which have been found unsuitable because of an erroneous subjective assessment

Taking into consideration the limited diagnostic and prognostic potential of routine methods, we decided to initiate a scientific clinical study aimed at searching and determining the effectiveness of additional objective methods for assessing the viability and functional state of the donor liver during preservation and in the early post-transplant period. Based on the analysis of the literature and the available technological potential of the clinic, interstitial microdialysis with the assessment of glucose metabolism parameters has been chosen as the study method.

## Materials and Methods

### Study design

**Figure 1.**
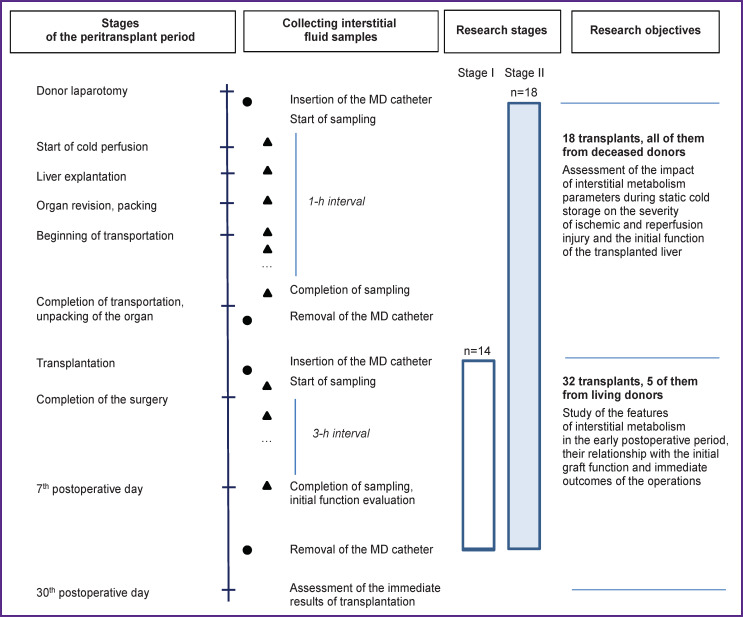
Design and objectives of the study Here: MD — microdialysis

The work was carried out in the Laboratory of New Surgical Technologies and subdivisions of the Surgery and Transplantation Center of the Burnazyan Federal Medical Biophysical Center of FMBA of Russia. It was a two-stage observational study (Figure 1); its results did not affect clinical decision making. The results were evaluated retrospectively. The analyzed transplants were not performed sequentially, the involvement of new cases was determined by the availability of informed consent of the patients, as well as a number of technical and organizational aspects not related to the demographic and clinical characteristics of the donors or recipients.

At stage I (until June 1, 2020), interstitial metabolism was studied only in the early postoperative period: from the completion of transplantation to the end of the 7th postoperative day. This part of the work included 14 observations, in 5 cases of which the right lobe of the liver from a living donor was transplanted.

At stage II (from June 1, 2020 to March 1, 2022), the period of sample collection and analysis of the composition of interstitial fluid was extended and lasted since the beginning of graft explantation, during the period of static cold storage (SCS) and transportation of the donor organ, as well as during the first week after surgery. There were 18 observations at stage II, all the organs were obtained from deceased donors.

In this way, 32 recipients were monitored for the parameters of interstitial metabolism after transplantation (27 grafts were obtained from deceased donors, 5 transplants — from living donors). The analyses of the observations were put together in order to study the dynamics of the interstitial concentrations of glucose, lactate, pyruvate, and glycerol in the early postoperative period, to establish their relationship with the initial function of the grafts (assessed on the 7th day), as well as with the immediate outcomes of operations (assessed on the 7th and 30th days).

To study the impact of the parameters of interstitial metabolism during SCS on the severity of ischemic and reperfusion injury (IRI) and the initial function of the transplanted liver, 18 cases were analyzed separately, in which the study was also carried out during SCS and donor organ transportation.

Early allograft dysfunction was defined according to criteria by Olthoff et al. [5]: the level of aspartate aminotransferase (AST) or alanine aminotransferase (ALT) is greater than 2000 U/L in the range from 24 h to 7 days after transplantation; and/or INR≥1.60 on the 7th day after transplantation; and/or total bilirubin concentration ≥10 mg/dL (≥171 μmol/L) on day 7 post-transplant. The transplants with an irreversible form of EAD, lost (retransplantation or death of the recipient) without restoration of the function during the first month after surgery for causes not related to surgical complications or acute rejection were referred to PNF.

**Figure 2.**
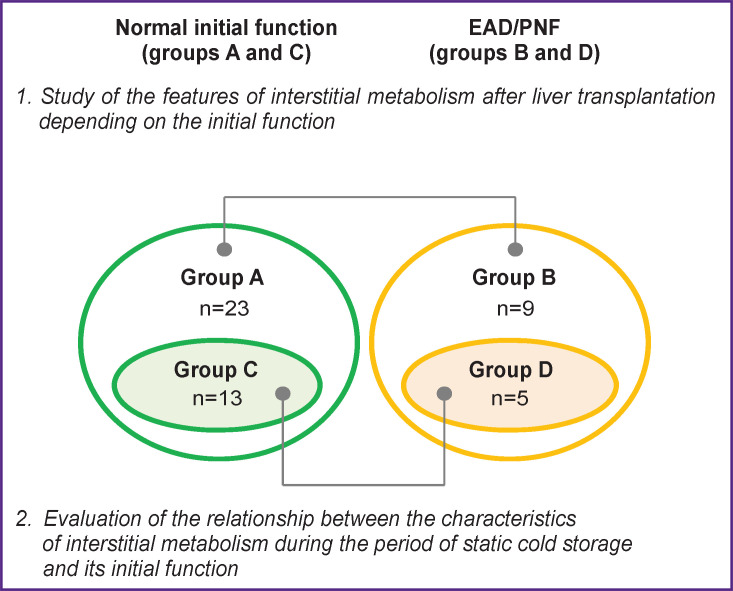
Distribution of observations in the groups depending on the aims of the study

To deal with each of the issues (see Figure 1), all the observations were retrospectively divided into groups depending on the initial graft function. (Figure 2). In the first part of the analysis, group A included cases of uncomplicated course (n=23); group B (n=9) included cases in which one of the following conditions developed in the first week after transplantation: PNF (n=1), EAD (n=8). In one case, EAD was secondary and developed against the background of a rare intraoperative complication — malignant hyperthermia, in another case, by the end of the second postoperative day, thrombosis of the hepatic artery developed on the background of EAD.

Out of 18 cases included in the second part of the analysis, 13 patients had an uneventful postoperative course (group C), EAD was diagnosed in 5 recipients (group D), and no cases of PNF were noted.

### Characteristics of recipients and donors

32 patients (21 men and 11 women) aged 38 to 68 years (the median was 53 years) were transplanted. All the procedures were primary. The indications for surgery were liver cirrhosis (LC) caused by viral hepatitis (n=12, of which 10 cases were HCV, 2 cases were HDV), hepatocellular carcinoma associated with LC of viral etiology (n=8), cholestatic liver diseases (n=3), unresectable liver alveococcosis (n=3), LC as a result of non-alcoholic steatohepatitis (n=2), alcoholic LC (n=2), LC of unknown etiology (n=2). Severity assessment by the MELD score varied from 7 to 40 points (the median was 15).

In 5 cases, the graft of the right lobe of the liver was obtained from living-related donors (3 men and 2 women) aged 22 to 49 years (the median was 32 years). Graft weight varied from 732 to 1130 g (median 885 g), the graft-recipient weight ratio ranged from 1.15 to 1.41% (the median was 1.25%).

In 27 cases, an organ for transplantation was obtained from deceased donors (18 men and 9 women) with established brain death, resulted from cerebrovascular accident (n=21) and severe traumatic brain injury (n=6). Donor age ranged from 20 to 67 years (the median was 48 years). The mean time spent on mechanical ventilation before retrieval was 3 days (from 1 to 6). In all the cases, hemodynamics was stable, without hypotensive and/or asystolic episodes. The results of the laboratory tests immediately before procurement were as follows: sodium — 147 (129–163) mmol/L, AST — 48 (14–200) U/L, ALT — 38 (7–110) U/L, total bilirubin — 10.2 (2.0–16.6) μmol/L. The median cold ischemia time (CIT) of the organs obtained from deceased donors was 9.0 (from 3.6 to 13.5) h. In 21 cases, aircraft transport was used for transportation. The CIT in living-donor liver transplants ranged from 30 min to 1 h 20 min (the median was 45 min).

### Installation of a microdialysis catheter, collection and analysis of interstitial fluid samples

The principle of microdialysis and its application in liver transplantation are known and described earlier [6-8], as well as in our previous work [9].

When an organ was procured from a deceased donor, a microdialysis catheter was placed in segment IV of the liver to obtain interstitial fluid samples immediately after sternolaparotomy and the initial stage of liver mobilization (crossing the round, falciform, right and left triangular ligaments) on the preserved blood flow. The catheter was fixed with a ligature to the falciform ligament, filled with perfusion fluid, and connected to a microdialysis pump. The pump was placed inside a sterile surgical glove, which was hermetically tied with a ligature and attached to the surgical drapes. 20 min after perfusion started, a sterile microtube was placed in the catheter port to collect interstitial fluid. Changing microtubes (i.e., obtaining individual samples of interstitial fluid) was performed once an hour until the organ was delivered to the operating room of the transplantation center. The feed rate of perfusate into the catheter was 0.3 μl/min.

After explantation, the organ was packed in the standard manner in three sterile plastic bags, with a microdialysis catheter sequentially passed through the node of each of them. When packing was completed, the sections of the catheter with a port for microtubes and connecting a microdialysis pump became non-sterile. The packed organ was placed in a transport isothermal container. Before unpacking the donor organ, the outer non-sterile part of the catheter was cut off; then unpacking was performed in the traditional way. Before starting the graft processing, the microdialysis catheter placed at the time of excision was removed.

A catheter for collecting samples of interstitial fluid in the early postoperative period was placed in the graft parenchyma immediately before suturing the surgical wound, brought out through the counteropening to the anterior abdominal wall and fixed to the skin.

In case of transplantation from a living donor at the stage of donor right-sided hemihepatectomy, after liver mobilization, before parenchyma splitting, a microdialysis catheter under ultrasound control was placed at the border of segments VII and VIII and fixed with a ligature to a fragment of the right triangular ligament.

After transplantation, the microtubes were changed every 3 h until the 7th postoperative day. During the study, the microdialysis pump was placed and fixed on the anterior abdominal wall of the patient. After sampling was completed, the microdialysis pump was turned off, and the catheter ports were closed. The catheter was removed on the 10–14th postoperative day, then a control abdomen ultrasound was performed.

Microdialysis catheters for hepatic tissue (61 Hepatic Microdialysis Catheter), Perfusion Fluid T1, 106 Microdialysis Pump, syringes for microdialysis pump (106 Syringe), and special microtubes with a volume of 200 μl were used to obtain interstitial fluid samples. The composition of interstitial fluid samples was determined 2 times a day on the ISCUS Clinical Microdialysis Analyzer using Reagent Set B. All the equipment and consumables were manufactured by CMA Microdialysis AB (Sweden) and have a registration certificate for a medical device No.FSZ 2007/00697 dated November 28, 2007, valid in the Russian Federation. The results of the study were digitized.

### Statistical data processing

Quantitative variables were presented as the median values, with additionally indicating either the minimum and maximum values, when it was them that represented clinical significance, or giving the interquartile range absolute frequencies indicated qualitative features. The significance of differences in quantitative and qualitative variables in two independent samples was determined using the nonparametric two-tailed Mann–Whitney test and two-tailed Fisher’s exact test, respectively. Differences were considered statistically significant at p<0.050. When calculating the odds ratio of a binary outcome, a 95% confidence interval (CI) was also indicated. To find the boundary values of quantitative parameters, ROC analysis was performed, sensitivity and specificity were calculated. Calculations were performed using the Statistica 12 software package (StatSoft Inc., USA).

## Results

The main characteristics of the recipients, donors, and transplants were matched by comparing groups A and B (Table 1). By the end of the first postoperative day, patients with EAD (group B) demonstrated a statistically significantly greater need for prolonged mechanical ventilation (more than 24 h), as well as a higher MELD score calculated from the results of laboratory tests obtained one day after surgery, compared with the observations in group A (normal initial graft function). The statistically significant difference in the aminotransferase levels is a consequence of the used criterion for the group formation, which includes the maximum AST/ALT levels during the first day after liver transplantation. In the vast majority of cases, it was precisely by the end of the first postoperative day when the peak values of these parameters were recorded and it may be considered as the quantitative assessment of the IRI severity of the graft. In spite of the formal absence of statistically significant differences, group B showed a clear trend towards a greater need for continued vasopressor support, and to higher levels of arterial lactate.

**Table 1:** Characteristics of donors, recipients, features of the transplants and their outcomes in the studied groups

Parameters	All observations (n=32)	Group A (n=23)	Group B (n=9)	pA–B	Group С (n=13)	Group D (n=5)	pC–D
***Characteristics of the recipients, donors, transplants***							
Recipient’s age (years)	53 [43; 58]	53 [46; 60]	48 [41; 58]	0.483	53 [52; 61]	56 [39; 58]	0.703
Class C by the Child–Pugh score (n)	10	8	2	0.681	5	1	0.615
MELD (points)	14 [12; 17]	15 [12; 17]	13 [12; 17]	0.711	13 [11; 17]	12 [11; 13]	0.289
Deceased donor (n)	27	19	8	1.000	13	5	1.000
SCS duration (h)	9.0 [7.8; 11.0]	8.7 [7.8; 10.7]	10.0 [8.3; 11.0]	0.328	8.3 [8.0; 10.3]	9.0 [7.6; 11.0]	0.924
Thermal ischemia (min)	40 [33; 45]	38 [32; 50]	45 [39; 45]	0.549	39 [33; 55]	45 [42; 45]	0.679
Reperfusion syndrome* (n)	5	2	3	0.121	2	1	1.000
Operation duration (h)	7.9 [7.0; 8.8]	7.5 [7.0; 8.3]	8.5 [7.0; 11.0]	0.145	7.8 [7.0; 8.1]	8.5 [7.0; 9.5]	0.443
RBC transfusion (ml)	305 [0; 640]	300 [0; 640]	600 [0; 930]	0.536	300 [0; 630]	0 [0; 600]	0.775
FFP transfusion (ml)	2345 [1900; 3070]	2480 [1920; 3070]	2140 [1790; 2990]	0.321	2350 [1880; 3040]	2260 [1600; 3200]	0.849
***24 h after transplantation***							
MLV longer than 24 h (n)	4	0	4	0.004	0	1	0.278
Vasopressor support (n)	7	3	4	0.076	2	2	0.533
AST (U/L)	736 [311; 2077]	389 [289; 811]	2728 [2213; 5254]	<0.001	750 [309; 908]	2728 [2098; 3284]	<0.001
ALT (U/L)	550 [289; 1237]	419 [246; 851]	1806 [1298; 2024]	<0.001	599 [387; 1041]	1298 [936; 1806]	0.075
Lactate (mmol/L)	1.7 [1.2; 2.3]	1.6 [1.2; 2.1]	2.2 [1.6; 3.9]	0.074	1.7 [1.4; 2.1]	1.9 [1.9; 2.9]	0.336
MELD after 24 h (points)	14 [11; 20]	14 [10; 17]	25 [20; 29]	<0.001	14 [10; 14]	20 [20; 29]	0.059
***7th postoperative day***							
AKI (RIFLE≥I) (n)	12	5	7	0.006	0	3	0.012
RRT (n)	7	2	5	0.010	1	3	0.044
Total bilirubin (μmol/L)	38 [21; 52]	32 [21; 50]	44 [28; 131]	0.126	36 [21; 51]	44 [28; 123]	0.441
INR	1.12 [1.09; 1.21]	1.11 [1.08; 1.15]	1.30 [1.11; 1.49]	0.071	1.11 [1.05; 1.15]	1.30 [1.11; 1.30]	0.267
Functioning transplants (n)	29	23	6	0.017	13	5	1.000
***Outcomes on the 30th postoperative day***							
Functioning transplants (n)	26	22	4	0.003	13	4	0.278

Note: * decrease in the mean arterial pressure by more than 30% below the basal level, exceeding 1 min in duration and developing within the first 5 min after liver transplant reperfusion. SCS is static cold storage; FFP is fresh frozen plasma; MLV is mechanical lung ventilation; AKI is acute kidney injury; RRT is renal replacement therapy; INR is an international normalized ratio. Quantitative data are presented as median, minimum and maximum values.

Assessment of the renal function of the recipients by the end of the first postoperative week demonstrated a statistically significantly higher frequency of episodes of acute renal injury (“Injury” and higher severity according to the RIFLE classification [10]) and a need for renal replacement therapy (continuous venovenous haemodiafiltration) in patients with EAD/PNF (group B). During the first postoperative week, three grafts were lost: on the 1^st^, 5^th^, and 7^th^ days after surgery due to PNF, hepatic artery thrombosis, and progressive multiple organ dysfunction syndrome (MODS), as well as severe EAD after the development of malignant hyperthermia in the intraoperative period. All the early graft losses occurred in group B. By the 30^th^ postoperative day (i.e. over the next three weeks), three more grafts were lost: two in group B (on the 22^nd^ and 26^th^ days as a result of the death of the recipients because of progressive MODS due to sepsis) and one case in group A (because of death on the 15^th^ day after transplantation in a patient with prior COPD and pneumosclerosis due to the progression of respiratory insufficiency and development of cardiovascular insufficiency, which required veno-arterial extracorporeal membrane oxygenation).

Due to the fact that the observations of groups C and D (the results of their comparative analysis are also given in Table 1) were included, respectively, in groups A and B, the trends described above were also observed at their matching. At the same time, the statistical significance of differences in a number of parameters, which was established at the comparison of group A and group B, was not achieved due to a smaller number of observations. During the first postoperative week, no graft losses occurred in groups C and D. In group D, one graft was lost on the 26^th^ day because of the patient’s death from progressive MODS due to sepsis.

Thus, in the cohort under consideration, the incidence of EAD was 25% (8/32), PNF was 3% (1/32); by the end of the first month after transplantation, 81% of recipients (26/32) were alive. In 5 of 6 cases of the loss of a transplanted liver, it occurred against the background of EAD/PNF. The odds ratio of graft loss in the development of early dysfunction, including PNF, was 27.5 (95% CI: 2.5–302.2).

### Interstitial glucose metabolism in the transplant during the first week after liver transplantation (32 observations: groups A and B)

**Figure 3.**
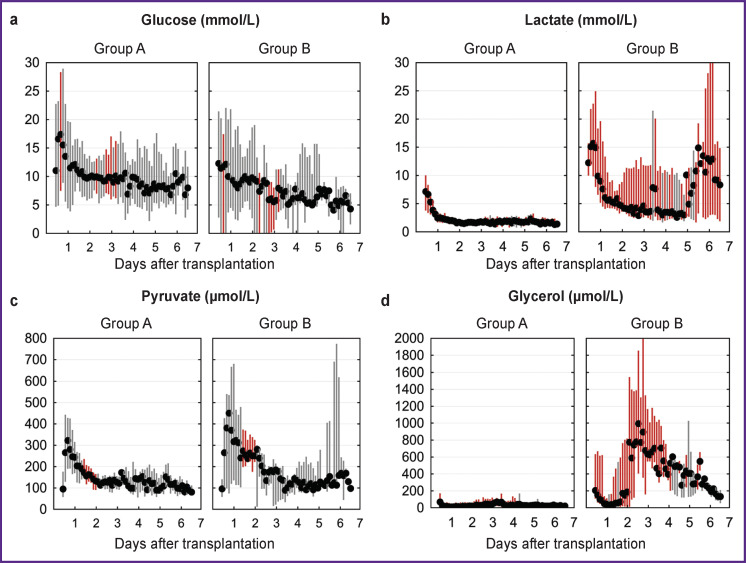
Dynamics of the interstitial concentrations of glucose (a), lactate (b), pyruvate (c), and glycerol (d) during the first 7 days after liver transplantation. Measurement interval — 3 h Black markers are the median parameter values. Vertical lines are their interquartile range. Statistically significant differences in values are indicated in red (p<0.050)

The dynamics of interstitial concentrations of glucose, lactate, pyruvate, and glycerol is shown in Figure 3. General patterns that do not depend on the graft function include:

moderately increased, compared with normal values for capillary blood, interstitial glucose concentration during the first day tending to decrease to 5–10 mmol/L during the 2^nd^–3^rd^ postoperative day;

the maximum levels of lactate registered during the first hours after the operation and its subsequent decrease;

an increase in the concentration of pyruvate in the first hours after the operation, reaching peak values after 18–24 h and then decreasing to 100–200 μmol/L.

Higher concentrations of lactate (compared to the smooth course of the postoperative period) in the first measurement 3 h after reperfusion — 12.3 [10.1; 15.6] mmol/L versus 7.2 [3.9; 9.9] mmol/L (p=0.003), and after 24 h — 6.1 [4.2; 13.2] mmol/L versus 2.4 [1.7; 2.8] mmol/L (p<0.001), with subsequent retention of statistically significant differences throughout the entire observation period, should be considered the most important feature of the dynamics of interstitial concentrations of the studied parameters in group B, i.e. with EAD development (Figure 3 (b)).

The ROC analysis determined the threshold value of interstitial lactate concentration to be 8.8 mmol/L. The use of the threshold value for the diagnosis of EAD provides 89% sensitivity, 65% specificity (the area under the ROC curve is 0.85).

Statistically significant differences in interstitial glycerol concentrations (Figure 3 (d)), which persist during the first 4 days, can be considered as a marker of cytolysis. With the development of EAD/PNF, 3 h after reperfusion, its concentration was 205 [118; 625] μmol/L; with normal initial function of the grafts, it was 69 [54; 162] μmol/L (p=0.008); 24 h after reperfusion it was 39 [25; 155] μmol/L and 17 [9; 54] μmol/L, respectively (p=0.031). The subsequent increase in glycerol concentration, observed in group B from the beginning of the second day, is probably not associated with the ongoing IRI, but it occurs due to the “iatrogenic” nature of this substance, and the addition of parenteral nutrition to the intensive care plan, which included fat emulsions containing glycerol. The decrease in the interstitial glycerol concentration, which began on the 4^th^–5^th^ postoperative day, chronologically coincided with the termination of parenteral nutrition. None of the patients in group A received parenteral nutrition during the first week after transplantation.

### Interstitial glucose metabolism in the liver of a deceased donor during static cold storage (18 observations: groups C and D)

**Figure 4.**
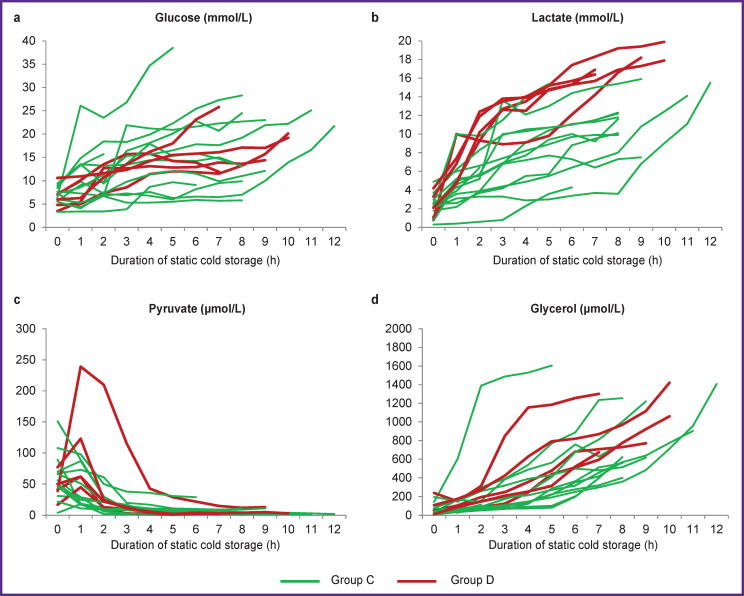
Dynamics of the interstitial concentrations of glucose (a), lactate (b), pyruvate (c), and glycerol (d) during static cold storage. Measurement interval — 1 h

In all the cases, the conditioning of deceased donors, procurement, preservation and transportation of donor organs were carried out by one surgical team in compliance with a single technology, the same criteria for assessing the quality and suitability of a donor liver for transplantation were used. Despite these facts, interstitial concentrations of the studied substances throughout SCS were ranged widely: glucose varied from 3.3 to 38.5 mmol/L, lactate — from 0.3 to 19.9 mmol/L, pyruvate — from 1 to 239 μmol/L, glycerol — from 6 to 1606 μmol/L. The universal trends were an increase in the concentrations of glucose (Figure 4 (a)), lactate (Figure 4 (b)) and glycerol (Figure 4 (d)), a decrease in the concentration of pyruvate (Figure 4 (c)), however, the dynamics of changes varied within the observations, so each case is shown as separate curves.

Additional analysis was performed to identify possible pre-transplant donor factors that could be associated with the initial function of the grafts: studying the demographic characteristics of deceased donors, the results of laboratory tests immediately before the procurement, the features of vasopressor and inotropic support, the type of transport, the results of retrospective histological examination, and also the parameters of interstitial glucose metabolism immediately before the start of *in situ* cold perfusion and at the end of SCS, the areas under the “concentration–SCS duration” curves (Table 2).

**Table 2:** Comparative analysis of the characteristics of deceased donors and parameters of interstitial glucose metabolism during static cold storage (observations of stage II of the study)

Parameters	Group С (n=13)	Group D (n=5)	p
***“Traditional” characteristics***			
Age (years)	50 (26–67)	51 (42–68)	0.924
Female (n)	7	5	0.114
Brain death resulted from CVA (n)	12	4	0.490
MLV duration before removal (days)	2 (1–4)	3 (2–4)	0.289
АSТ (U/L)	33 (14–200)	32 (10–61)	0.775
АLТ (U/L)	26 (13–110)	24 (10–35)	0.289
Total bilirubin (μmol/L)	10 (3–21)	10 (7–16)	0.775
Creatinine (μmol/L)	122 (65–893)	101 (44–382)	1.000
Sodium (mmol/L)	149 (138–163)	145 (136–159)	0.566
Dopamine only (n)	1	0	1.000
Norepinephrine only (n)	7	2	1.000
Dopamine and norepinephrine (n)	4	1	1.000
Dopamine dose (μg/kg/min)	5.0 (2.8–10.0)	0.5	—
Norepinephrine dose (ng/kg/min)	320 (10–820)	650 (620–1500)	0.088
Aircraft transport (n)	10	3	0.583
Static cold storage duration (h)	8.3 [8.0; 10.3]	9.0 [7.6; 11.0]	0.924
Donor Risk Index [11] (points)	1.70 (1.28–1.98)	1.62 (1.49–1.91)	1.000
Macrovesicular steatosis (n)	5	3	0.608
Steatosis area (%)	10 (5–20)	10 (5–50)	0.786
***Parameters of interstitial glucose metabolism***			
Interstitial concentrations (before cold perfusion):			
glucose (mmol/L)	7.2 (3.3–9.4)	6.0 (3.5–10.6)	0.387
lactate (mmol/L)	2.5 (0.3–4.9)	2.1 (0.8–4.2)	0.849
pyruvate (μmol/L)	56 (4–151)	41 (17–77)	0.289
glycerol (μmol/L)	32 (6–196)	56 (12–239)	0.288
Interstitial concentrations at the end of SCS:			
glucose (mmol/L)	15.7 (5.8–38.5)	19.2 (11.9–25.8)	0.924
lactate (mmol/L)	11.8 (4.3–15.9)	17.9 (16.4–19.9)	<0.001
pyruvate (μmol/L)	5 (1–31)	3 (2–13)	0.387
glycerol (μmol/L)	623 (114–1606)	1060 (673–1421)	0.208
Area under the “SCS–interstitial concentration” curve:			
glucose (mmol/L·h)	109.3 (25.7–182.2)	106.1 (92.7–160.3)	1.000
lactate (mmol/L·h)	61.6 (8.4–102.3)	99.5 (87.5–140.4)	0.002
pyruvate (μmol/L·h)	121 (69–322)	146 (89–711)	0.387
glycerol (μmol/L·h)	3447 (149–5883)	4287 (1878–6834)	0.117

Note: CVA is acute cerebrovascular accident; MLV is mechanical lung ventilation; SCS is static cold storage. Quantitative data are presented as the median and interquartile range or minimum and maximum values.

The comparative analysis of the groups showed statistically significant differences only for the values of interstitial lactate concentration and the area under the “lactate concentration–SCS duration” curve. The subsequent ROC analysis established the boundary values of 15.4 mmol/L and 76.1 mmol/L**·**h for these parameters, the excess of which, with 100% sensitivity in both cases and a specificity of 77 and 85%, respectively, can be used to predict initial graft dysfunction.

## Discussion

The function of the transplanted liver in the first hours and days after surgery is the leading factor in determining both the intensive care plan and the prognosis for a recipient. The results of routine diagnostic tests do not always reflect the real severity and reversibility of ischemia-reperfusion injury of the graft. The use of efferent methods, intensive infusion and transfusion therapy may reduce the information value of the laboratory methods. Supplementing the standard set of studies with the determination of the parameters of interstitial glucose metabolism in dynamics makes it possible to specify a severity level and estimate the IRI reversibility of a liver graft. In addition, high sensitivity of the method in detecting ischemic changes in some cases can significantly accelerate the diagnosis of complications associated with compromised blood flow through the graft hepatic artery.

The results of monitoring the parameters of interstitial metabolism in a liver transplant in the early stages after transplantation, obtained by our group, are the first and currently the only ones in Russian clinical practice. Although a number of foreign centers started the use of interstitial microdialysis in transplantation about 15–20 years ago, the total number of conducted observations has been small. In this regard, there is currently no unified view on the diagnostic and prognostic significance of individual parameters and the dynamics of their changes. Nevertheless, the results of this study do not contradict and are highly consistent with the previously published works by Nowak et al. [6], Waelgaard et al. [7], Haugaa et al. [8, 12].

Challenges in predicting early dysfunction of a transplanted liver, known to many specialists, are caused by the multifactorial nature of this phenomenon. Not only early diagnosis, but also the establishment of etiology are important when the initial graft function is impaired. As we see it, the broad concept of “early graft dysfunction” should be divided into:

primary EAD, the causes of which are the specific characteristics of the donor (age, cause of death, concurrent diseases and conditions, hemodynamic parameters), the donor organ (laboratory evaluation of the function, ultrasound characteristics, steatosis severity), as well as preservation (quality of *in situ* perfusion, preservation solution, the duration of graft preservation, and, possibly, transportation conditions);

secondary EAD, caused by the initial severity of the recipient’s condition, specific characteristics of surgery and anesthesia during operations and intraoperative complications.

Graft dysfunction that develops in the early postoperative period due to surgical complications (e.g., hepatic artery thrombosis), rejection, infections, cardiovascular and respiratory complications, should be considered as a separate condition.

The methods for assessing the quality of the deceased-donor liver used in real clinical practice are, to a certain degree, subjective, and this is what complicates the differential diagnosis of primary and secondary EAD. Normothermic perfusion with oxygenated erythrocyte-based media becomes a solution to this problem and, in the vast majority of cases, makes it appropriate to use grafts of subjectively non-ideal (marginal) quality, excluding the risk of severe primary EAD and PNF [13]. It should be noted that, despite the active development of this area over the past decade, the use of normothermic perfusion is associated with an increasing complexity of the peritransplant process, substantial financial costs, and increased demand for high-skilled professionals. In Russia, despite the high relevance of this method, it seems doubtful to be used not only in routine practice, but also in the format of a large clinical trial in the coming years.

It is these circumstances, as well as a unique feature of the Coordination Center for Organ Donation of the Burnazyan Federal Medical Biophysical Center (most of the organs are transported to the clinic from donor hospitals located in other regions of the country at a distance of up to 3500 km, which is associated with the inevitable prolongation of SCS up to 8–12 h) that have become decisive for initiating and conducting stage II of this study, i.e. studying the features of interstitial glucose metabolism in the deceased-donor liver during SCS, assessing of their relationship with the IRI severity and the initial graft function. In the analysis of previously published works, we have found only one similar clinical study performed by Silva et al. [14], consisting of a series of 15 transplantations. The fundamental methodological difference of our group’s approach is the continuous collection of the interstitial fluid samples of a donor organ from the moment of graft explantation until delivery to the transplantation center. Silva et al. did not perform sampling during the transportation period, limited to the measurements at the donor hospital (before organ packing) and at the back-table stage. Nevertheless, the results obtained are completely consistent with each other. The dynamics of changes in the concentrations of glucose, lactate, and pyruvate confirms the fact that, under SCS, the processes of glycogenolysis and anaerobic glycolysis run in parallel in the deceased donor liver, and the accumulated concentration of lactate may reasonably be considered as a predictor of primary EAD.

Despite the described advantages of the method of interstitial microdialysis, the practicability of its routine use in clinical practice is debatable. The retrospective nature of the results of the study of the transported donor organ is supposed to be the main disadvantage since the analysis of the composition of microsamples of the interstitial fluid is performed on the stationary equipment at the transplantation center. This either excludes the use of the results at the time of making a decision on the suitability of the organ for transplantation and the start of the operation in the recipient, or requires additional time to conduct the study after the delivery of the organ to the clinic, which causes a delay in the start of the operation until the results are obtained and, as a result, the SCS duration is extended by up to 2–3 h. Moreover, studying interstitial metabolism requires specialized equipment (analyzer, microdialysis pumps) and disposable consumables. Compared to the technology of isolated normothermic perfusion, their cost is significantly lower (by a factor of 10 or more), however, it demands leveraging additional financial resources: the study only during the SCS period accounts for about 70 thousand rubles, monitoring extension in the early postoperative period requires about 100 thousand rubles (per transplant, as at 2021).

Obviously, addressing these challenges rests exclusively in the area of technical implementation of the method for obtaining and analyzing the composition of interstitial fluid samples. We consider it to be promising to shift from the photocolorimetric method of measuring metabolite concentrations in the microsamples of interstitial fluid to the use of flow-through electrochemical sensors and obtaining the results of the analysis in real time. The development of a specialized monitoring module coupled with such sensors will provide sending data during transportation, and, if necessary, continuous measurement of other parameters (for example, organ temperature, external conditions of transportation).

**Figure 5.**
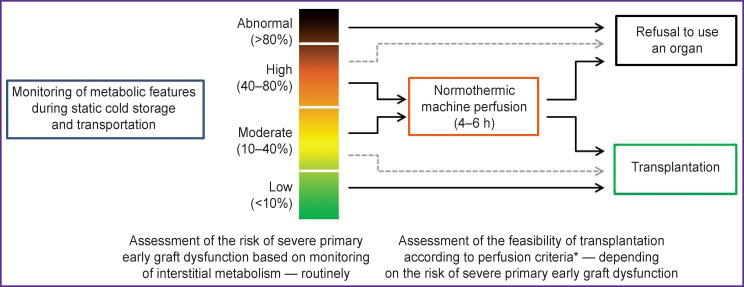
A novel concept for the assessment of donor organ viability, predicting the risk of primary early graft dysfunction and the feasibility of its transplanting to a human Solid arrows indicate the sequence of actions with the use of normothermic perfusion technology, dotted arrows — when this technology is not available; * the use of validated criteria is assumed, for example [15]

In our opinion, the creation of a portable medical device for monitoring the state of interstitial metabolism both during preservation and in the postoperative period for early diagnosis of functional disorders and vascular complications would provide adequate clinical studies in order to establish accurate diagnostic and prognostic criteria and be a decisive factor for the subsequent rapid and large-scale introduction of the interstitial microdialysis technology into the clinical practice of organ donation and transplantation. The opportunity of using the same device at all stages and for any options for static and perfusion preservation of organs provides formulation and proposal of a new concept for assessing their quality, predicting the risk of severe primary EAD and PNF (Figure 5). At the same time, the monitoring of interstitial metabolism should not be considered as an alternative to normothermic perfusion. In our opinion, the best approach is the availability of the both technologies in the toolbox of the organ donation services and transplantation centers.

It should be underlined that the assessment of the parameters of interstitial metabolism during the preservation period and obtaining additional objective data on the state of the donor organ can be carried out not only for the liver. This is confirmed by the findings in published works on monitoring the state of the kidney in the experiment [16, 17] and the lungs [18].

## Conclusion

The extension of the criteria for the suitability of donor organs for human transplantation is an inevitable process that has accompanied the entire history of clinical transplantation. The most efficient use of organs from deceased donors implies not only an increase in the number of transplants performed but also the achievement of satisfactory immediate and long-term results. This principle determines the extremely high relevance and need for search work and clinical studies of new technologies for the preservation and assessment of the viability of donor organs. One of the already ongoing activities is the development of methods and devices for isolated normothermic perfusion. Although, static cold storage has been and remains a reliable approach for organ preservation, the main disadvantage of which is the inability to monitor the organs’ condition. The findings of foreign studies, as well as the results of our own practice, allow us to rightly claim that the study of the parameters of interstitial metabolism of glucose (mainly lactate) will solve this problem.

Moreover, the use of interstitial microdialysis in the early post-transplant period gives an immediate (within a few hours after reperfusion) assessment of the initial graft function, allows to differentiate primary and secondary EAD, and, accordingly, have a chance to adjust the intensive care plan in a preventive mode. Continued monitoring during the first days after surgery can be considered as a method for early diagnosis of vascular complications.

Technical improvement of the process of collecting and analyzing samples of interstitial fluid and the creation of a medical device will be crucial for the further development and widespread implementation of the technology into clinical practice.
